# Synthesis of gold nanoparticles derived from mannosylerythritol lipid and evaluation of their bioactivities

**DOI:** 10.1186/s13568-019-0785-6

**Published:** 2019-05-07

**Authors:** Abdelmoneim Bakur, Yongwu Niu, Hui Kuang, Qihe Chen

**Affiliations:** 1 Department of Food Science and Nutrition, Zhejiang University, Yuhangtang Rd. 866, Hangzhou, 310058 China; 2 Department of Food Sciences and Technology, University of Kordofan, El Obeid, Sudan

**Keywords:** Mannosylerythritol lipids, Gold nanoparticles, Anticancer, Antioxidant activity, Antibacterial activity

## Abstract

In this study, we introduce a simple and green method for synthesis of gold nanoparticles (AuNPs) using microbial glycolipid mannosylerythritol lipid (MEL) produced from *Ustilago maydis* CGMCC 5.203 and to evaluate their biomedical activities. MEL was found 10.3 g/L using sunflower oil. The formation of MEL-AuNPs was verified using UV–visible spectrum, XRD, TEM, FTIR, SEM, and EDX. In the biomedical examinations, MEL-AuNPs demonstrated potential cytotoxicity against HepG2 cells, and IC_50_ values were found to be 100 and 75 µg/mL for 24 h and 48 h of exposure, respectively, which indicates its good performance against cancer cells. The IC_50_ value of MEL-AuNPs was found to be 115 and 124 µg/mL for DPPH and ABTS scavenging activities, respectively. The biosynthesized MEL-AuNPs significantly inhibited cell growth of pathogenic Gram-positive and Gram-negative bacteria. These findings indicated that MEL plays a crucial role in the rapid biofabrication method of metallic NPs possessed the potential of biomedical activities.

## Introduction

In recent years, gold nanoparticles (AuNPs) have gained considerable attention due to their wide range of potential applications including anticancer (Rajeshkumar 2016) antimicrobial (Dorosti and Jamshidi 2016), antioxidants (Muthuvel et al. 2014), and agriculture (Mahakham et al. 2016). Besides, they have widely used in applications of the biolabelling, photothermal therapy, tissue/tumor imaging, biosensors, drug delivery and detection of the pathogens (Ganesh Kumar et al. 2011). Nowadays, considerable attention has been paid to the green synthesis of nanoparticles through biosurfactants and biological substances, because of hazardous and toxic by-products are usually paired with chemical methods (Kasture et al. 2008; Kumar et al. 2010). As evident from earlier studies, biosurfactants can act as both reducing and stabilizing agents in the synthesis of NPs, such as lipopeptide biosurfactant, sophorolipids, and rhamnolipid (Kasture et al. 2008; Reddy et al. 2009; Priyadarshini et al. 2016). Synthesis of nanoparticles by biosurfactant is superior to the biological methods, due to the (Kiran et al. 2011). Previous researchers indicated that surfactants mediated nanoparticles might improve antibacterial activities, such as SDS, PVP 360, Tween 80 and CTAB have significantly improved the antibacterial activity for most bacteria species (Kvitek et al. 2008; Alkilany et al. 2009).

**Fig. 1:**
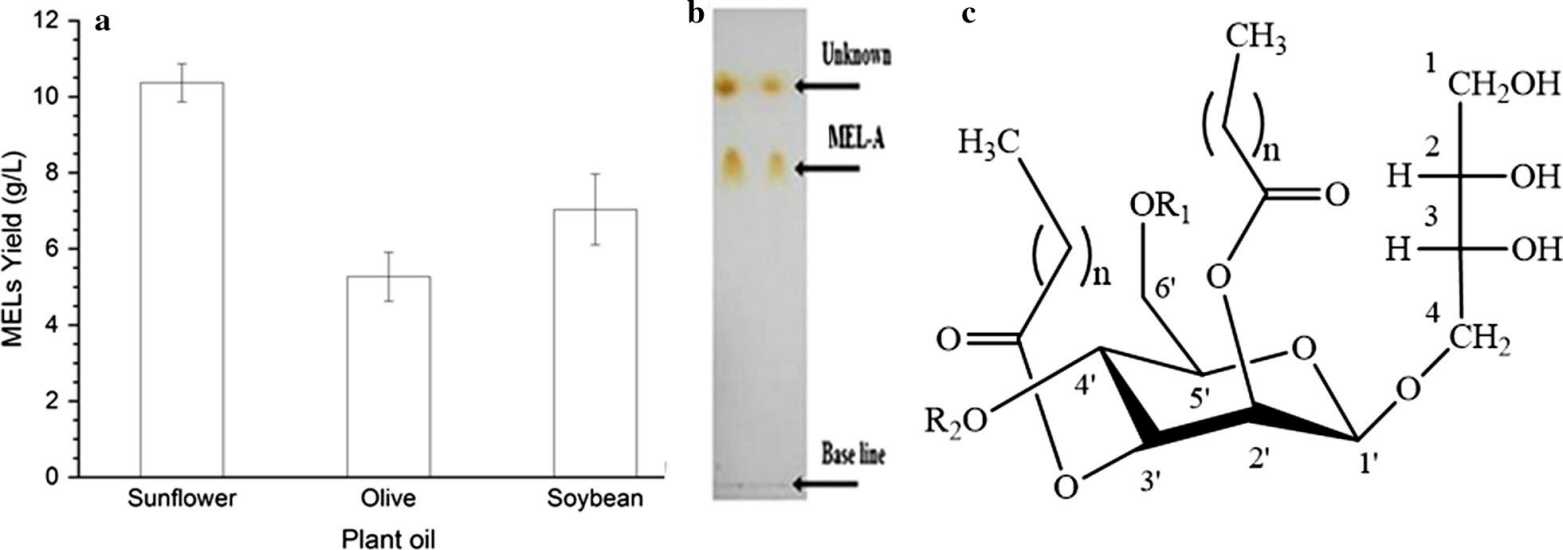
**a** Comparison of crude MEL production from different Carbone source; **b** analysis of MEL production by TLC; **c** structure of mannosylerythritol lipid (MEL)

Mannosylerythritol lipids (MELs, Fig. 1c) are microbial glycolipid biosurfactants comprised of 4-*O*-β-d-mannopyranosyl-d-erythritol and a fatty acid or an acetyl group acting as hydrophilic and hydrophilic moieties, respectively Kitamoto (2008). MEL has been exploited in many fields such as potent antimicrobial activity and interfacial properties (Morita et al. 2006). As well, MEL shows numerous bioactivities, such as, induce cells of apoptosis and differentiation of mouse melanoma, rat pheochromocytoma and extensively applied in food, pharmaceutical, cosmetic and environmental protection (Zhao et al. 1999; Wakamatsu et al. 2001; Fan et al. 2016).

The present work aimed to produce MEL from *Ustilago maydis* CGMCC 5.203 by resting cell fermentation under the limited nitrogen using different carbon sources. A further aim was to develop highly stabilized AuNPs using the produced MEL via one-step technique and verified by different techniques such as UV–vis absorption spectrum, TEM, SEM, EDX, FT-IR, and XRD (X-ray diffraction). Finally, the potential bioactivities of MEL-AuNPs in antibacterial, antioxidant, and cancer cell cytotoxicity were evaluated.

## Materials and methods

### Materials

All media components and chemicals were purchased from Sangon Biotech (Shanghai) Co., Ltd. (China). Other reagents and solvents used in this work were used as received. All reagents used were of the highest purity available.

## Microorganisms

*Ustilago maydis* CGMCC 5.203 was obtained from China General Microbiological Culture Collection Center. Moreover, this fungus was growing at 28 °C in liquid YEPS (1% yeast extract, 2% peptone, 2% sucrose). *U. maydis* CGMCC 5.203 was preserved in 50% glycerol (v/v) at − 80 °C. *Staphylococcus aureus* CCTCC AB91093 and *Escherichia coli* CCTCC AB91112 were obtained from China Center for Type Culture Collection (China, Wuhan).

### MEL production by resting cells cultivation

Seed cultures were prepared by inoculating cells previously grown on a YEPS, into 250 ml Erlenmeyer flasks containing 50 mL of a growth medium [0.1% (w/v) KH_2_PO_4_, 0.02% (w/v) citric acid, 0.04% (w/v) MgSO_4_·7H_2_O, 0.003% (w/v) FeSO_4_·7H_2_O, 0.06% (v/v) corn-steep liquor, 0.06% (w/v) urea, 5% (w/v) glucose and distilled water]. Then the cultures were incubated at 28 °C on a rotary shaker (120 rpm) for 3 days. After it, the cells were harvested by centrifugation at 3500 *g* for 15 min and washed twice with 0.9% NaCl solution under sterile conditions. The obtained cells were transferred into 250 mL Erlenmeyer flasks containing 50 mL of a fermentation medium, containing the same composition as the main culture except for glucose which was replaced by 1.0% (v/v) plant oil (sunflower, olive, and soybean oil). After that, the culture flasks incubated on a rotary shaker (120 rpm) at 28 °C for 8 days, unless otherwise indicated.

### Extraction and purification of MELs

At the end of fermentation, the culture broth containing MELs was extracted with an equal amount of ethyl acetate and centrifuged for 15 min at 4000 rpm. The organic layer was separated, evaporated, and crude mixtures of MELs were washed with methanol and cyclohexane to remove the residual oil. The crude MEL extracted was diluted with chloroform and then purified by silica-gel column chromatography using chloroform–acetone (Onghena et al. 2011). The purified MEL was confirmed by using thin layer chromatography (TLC) (Silica gel 60 F, chloroform: methanol: water = 65:15:2, v/v) as the solvent system. The FTIR spectrometer (Vector 22, Bruker, Germany) was used to verify various functional groups existed in MEL.

### Biosynthesis of gold nanoparticles

In the present study, mannosylerythritol lipid (MEL) was utilized as both the reducing and capping agents in the synthesis of gold nanoparticles (AuNPs). Briefly, 200 μL of MEL in 1 mL of methanol diluted to 10 mL of distilled water then added to 50 mL an aqueous solution of 3 mM HAuCl_4_ under magnetic stirring at 60 °C for 30 min. The pH value of the mixture was adjusted to 8 using 0.1 M of KOH. The change of color to purple visually observed the formation of AuNPs.

### Characterization of the biosynthesized MEL-AuNPs

The bioreduction of the gold ion by MEL was verified by UV–Vis spectrophotometer (UV-2550 Shimadzu, Japan) at a resolution of 1 nm in the wavelength of 300–800 nm. For further characterization, an aqueous solution of MEL-AuNPs was centrifuged at 14,000 rpm for 20 min. Repeated rinses were achieved to remove impurities and were lyophilized. The FTIR spectrum analysis was collected at the resolution of 4 cm^−1^ in the range of 500–4000 cm^−1^ region using Fourier transform infrared spectrometer model (Vector 22, Bruker, Germany). The crystalline nature of MEL-AuNPs was evaluated via Siemens X-ray diffractometer (XRD) analysis, where XRD patterns were measured by drop coated film of dried powder of MEL-AuNPs onto glass slides. The operation conditions were at a voltage of 45 keV and a current of 20 mA with Cu-Ka radiation as an X-ray source in the range of 20–80 at the 2-theta angle. The structural characterization of MEL-AuNPs was carried out by transmission electron microscopy (TEM) (JEM-1230, JEOL, Akishima, Japan). The extra sample was removed from the carbon-coated copper grid using the cone of a blotting paper and samples were placed on the carbon-coated copper grid to make a thin film of the sample, and then it was reserved in a grid box sequentially. Scanning Electron Microscopic (SEM) (TM-1000, Hitachi, Japan) analyzed the size of the synthesized AuNPs. Thin film samples were prepared on a carbon-coated copper grid by dropping the sample on the grid, and an excess solution was removed via a blotting paper. Then, the film on the SEM grid was allowed to dry by putting the grid under a mercury lamp for 5 min. The device was supplemented with an energy-dispersive X-ray spectrum (EDX) to verify the existence of AuNPs.

### In vitro assessment of biological activities of MEL-AuNPs

#### Antibacterial activity

The antibacterial activity of newly synthesized MEL-AuNPs was evaluated against Gram-positive *S. aureus* CCTCC AB91093 and Gram-negative *E. coli* CCTCC AB91112 by well diffusion technique (Srinivasan et al. 2001). Briefly, bacterial suspensions at concentrations (approximately 5 × 10^5^ CFU/mL) were uniformly spread on Mueller–Hinton Agar (MHA) plates. Three wells about 6 mm diameter were created in each of these plates using sterile borer. 50 µL of the biosynthesized MEL-AuNPs solution were added at various concentrations (25, 50 and 100 µg/mL) to the wells at aseptic conditions, and the tested plates were incubated at 37 ± 2 °C for 24 h. After it, the diameter of an inhibitory zone was measured. This study was carried out in triplicates.

#### Anticancer activity against HepG2 cell line

The cytotoxicity potential of MEL-AuNPs was investigated by MTT assay using human liver hepatoma HepG2 cells. The cells were procured from Shanghai Cell Bank of China and maintained in DMEM (Dulbecco’s Modification Eagle Medium) containing 10% fetal bovine serum (FBS) and 1% antibiotics solution (10^5^ U/L penicillin and 100 mg/L streptomycin) in a humidified incubator with 5% CO_2_ at 37 °C.

The HepG2 cells were placed into 96-well plates at a density of 1.25 × 10^5^ cells/well and incubated for 24 h. Afterwards, the cells were treated with different concentrations of MEL-AgNPs (10–150 µg/mL) and MEL, the proliferation activity of the cells was determined by adding 5.0 mg/mL MTT reagent after 24 h and 48 h of incubation. Finally, the absorbance was scanned at 570 nm in a microtitre plate reader (Thermo Electron Corp, Asheville, NC). The viability of HepG2 cells was expressed as a percentage of the control culture value, which was considered to be 100% viable.

#### Antioxidant capacity

DPPH and ABTS examinations were exploited to evaluate the total antioxidant capacity of the nanoparticle. The DPPH radical scavenging activity of MEL-AuNPs samples was determined using DPPH assay method described by Saratale et al. (2017). About 0.2 mL of different concentrations of MEL-AuNPs were mixed with 2 mL of DPPH solution. The mixture was vortexed and kept at 24 °C for half an hour in the dark. The absorbance of the reaction mixture was read spectrophotometrically at 517 nm using BHT as a standard.

For ABTS scavenging activity of MEL-AuNPs was evaluated following the method described by (Moldovan et al. 2016) with some modifications. The ABTS radical cation was produced by mixing an ABTS stock solution (7.0 µM) and potassium persulphate (2.45 µM) and allowing the mixture to react in the dark at ambient temperature for 16 h. The ABTS^.+^ solution was diluted with ethanol 80% (v/v) to an absorbance of 0.700 ± (0.05) at 734 nm on the microplate reader (Thermo Electron Corp, Asheville, NC). About 200 µL of the ABTS^.+^ solution was mixed with 20 µL of the MEL and MEL-AuNPs solution at different concentrations. After 6 min in the dark, the absorbance at 734 nm was measured using ascorbic acid as a standard. The absorbance of a blank solution (without samples) was also measured. The radical scavenging activity was expressed as the IC_50_ values. The Free radical scavenging percentages of DPPH and ABTS^+^ radicals by MEL-AuNPs were calculated according to the following formula:

$${\text{Free radical scavenging }}\left( \% \right)\, = \,\left[ {{{\left( {{\text{A}}_{\text{C}} \, - \,{\text{A}}_{\text{T}} } \right)} \mathord{\left/ {\vphantom {{\left( {{\text{A}}_{\text{C}} \, - \,{\text{A}}_{\text{T}} } \right)} {{\text{A}}_{\text{C}} }}} \right. \kern-0pt} {{\text{A}}_{\text{C}} }}} \right]\, \times \, 100$$

where A_C_ is the absorbance control of DPPH and ABTS in two different assays, while A_T_ is the absorbance of DPPH and ABTS free radical in the existence of Au nanoparticles at 517 nm and 734 nm, respectively.

### Statistical analysis

The data were expressed as the standard deviation (mean ± SD) obtained from at least three independent experiments. The statistical analysis was evaluated by one-way ANOVA test (SPSS 16) followed by Tukey’s HSD test (*p* < 0.05).

## Results

### Production and characterization of MELs

Figure 1a shows the production of MELs from different carbon sources by the resting cells biotransformation of *Ustilago maydis* CGMCC 5.203 under growth-limiting nitrogen conditions. After fermentation, extracted and purified by silica-gel column chromatography, then it was confirmed by using thin layer chromatography (TLC) (Fig. 1b). In this work, the total yield of MELs from sunflower, olive, and soybean oil were 10.36, 5.26, and 7.03 g/L, respectively (Fig. 1a).

### Visual observations and UV–vis absorption spectra (UV–vis) studies

**Fig. 2:**
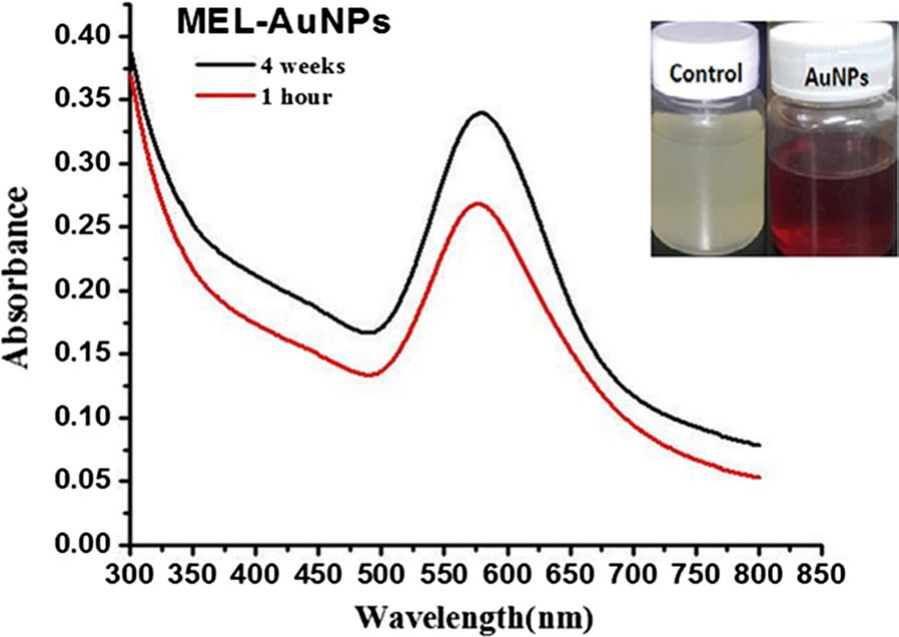
UV–Vis spectra of biosynthesized MEL-AuNPs using MEL

The reduction of HAuCl_4_ to AuNPs was successfully achieved after optimization of the experimental parameters, by the aqueous solution of 3 mM HAuCl_4_ and aqueous MEL solution, which employed as both the reducing and capping agents. The change of color to purple was visually observed. This step was followed by UV–vis absorption spectroscopy as shown in Fig. 2. The strong surface plasmon resonance (SPR) was located at 566 nm.

### Morphological characterization of biosynthesized MEL-AuNPs

**Fig. 3:**
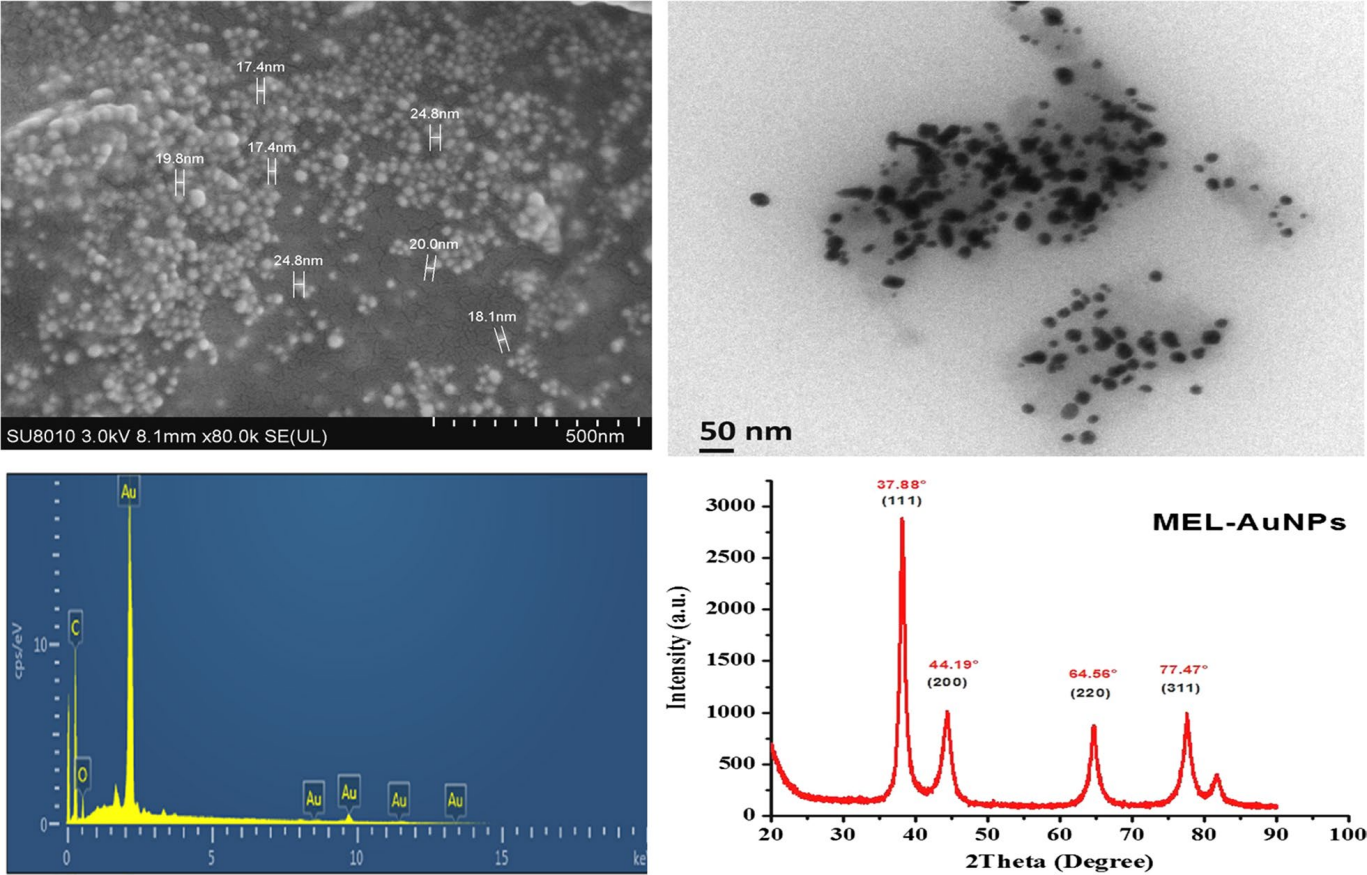
Characterization of biosynthesized AuNPs using MEL **a** SEM image; **b** TEM image; **c** EDX pattern; and **d** XRD pattern

SEM image of the biosynthesized MEL-AuNPs exhibited a spherical shape as shown in Fig. 3a. Besides, the EDX Spectrum analysis has demonstrated the absorption of strong Au signal (Fig. 3b). The absorption peak of Au was observed at 2.2 keV, which is typical of the crystalline nature of the AuNPs. Other signals corresponding to C, O have also appeared. Furthermore, TEM image of MEL-AuNPs (Fig. 3c) confirmed that the sample is mainly comprised of spherical and uniform in shape. Moreover, the X-ray diffraction patterns of the biosynthesized MEL-AuNPs are shown in Fig. 3d. The diffraction peaks of MEL-AuNPs are centered at 2*θ* = 37.88°, 44.19°, 64.56°, and 77.47° corresponding to the planes of (111), (200), (220) and (311), respectively.

### Fourier transformed infrared spectroscopy (FTIR)

**Fig. 4:**
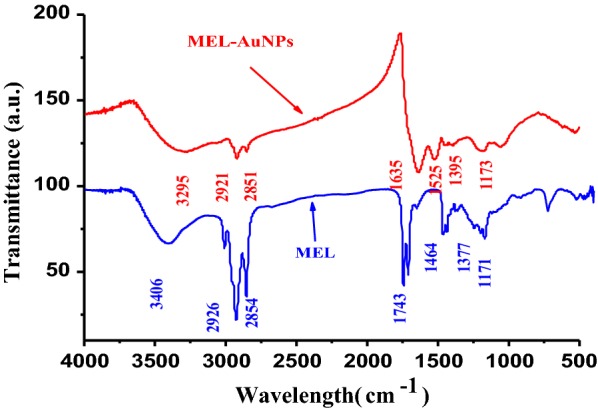
FTIR spectrum of MEL-AuNPs and MEL

The FTIR spectra illustrated the possible interactions between the MEL and AuNPs (Fig. 4). The FTIR spectrum of MEL displayed transmittance peaks at 3406, 2926, 2854, 1743, 1464, 1377 and 1171 cm^−1^ corresponding to (O–H), (C–H), (C=O), (C–H) and (C–O), respectively that implying the complex nature of MEL, Fukuoka et al. reported similar findings (Fukuoka et al. 2007). The broadband at 3275 cm^−1^ attributed to O–H stretching vibrations. The distinct absorption bands at 1395 cm^−1^ and 2921 cm^−1^ demonstrated the existence of a long fatty acid chain. The strong and broad bands at 2921–2854 cm^−1^ and 1395 cm^−1^ denote the presence of C–H stretching, matching to CH_2_ and CH_3_ groups of aliphatic chains. The absorbance bands at 1635 cm^−1^ and 1179 cm^−1^ indicate the presence of fatty acids.

### Antibacterial activity of biosynthesized MEL-AuNPs

**Fig. 5:**
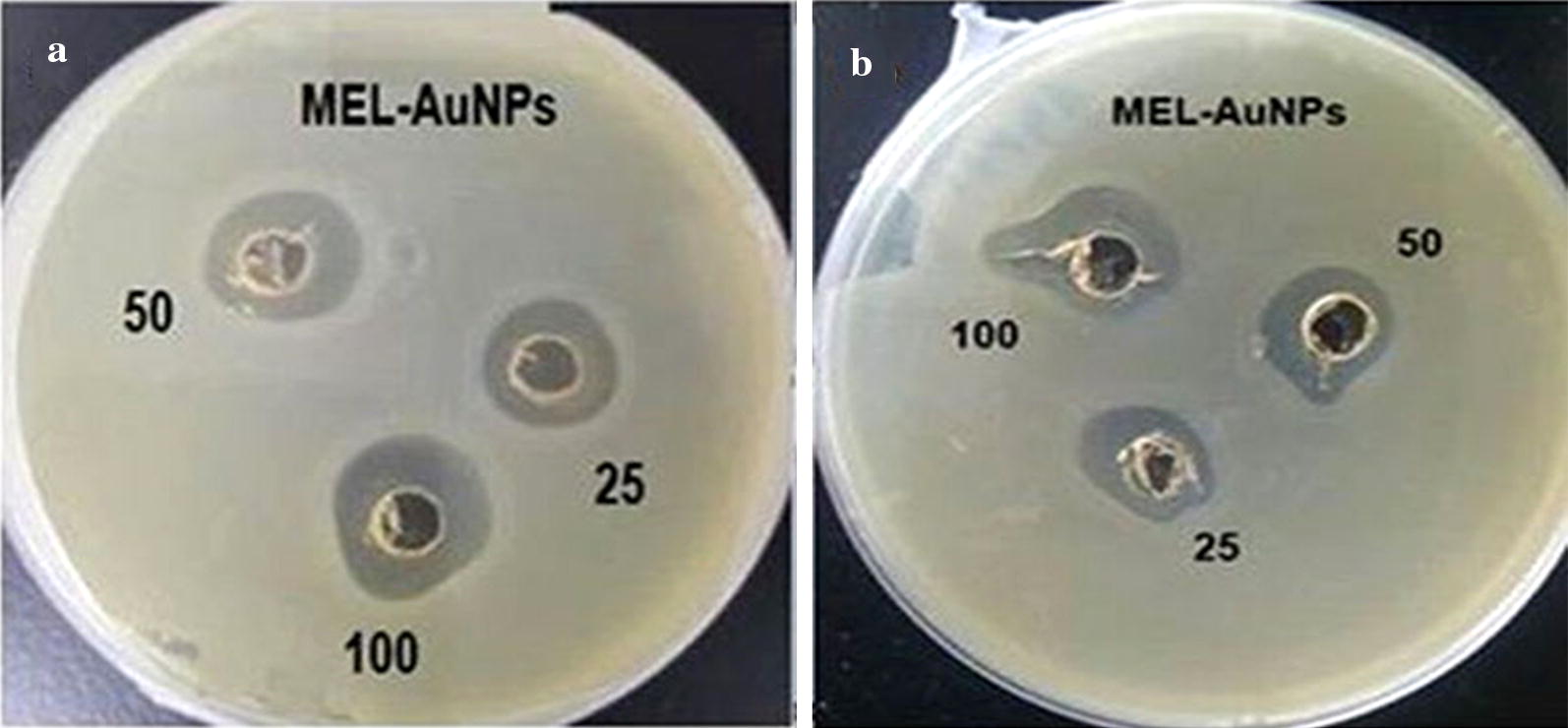
Antibacterial activity of biosynthesized MEL-AuNPs against **a** *Escherichia coli* and **b** *Staphylococcus aureus*

Figure 5 shows the antibacterial potential of freshly biosynthesized MEL-AuNPs against Gram-positive and Gram-negative bacteria at the concentration of 25, 50 and 100 µg/mL using well diffusion method, and results are presented in Table 1. MEL-AuNPs displayed a noticeable antibacterial effect against *E. coli* and *S. aureus* at the minimum concentration. The average inhibition zone of MEL-AuNPs against bacteria cells ranged from 12 to 17.6 mm.

**Table 1:** Antibacterial activity of biosynthesized MEL-AuNPs

Pathogenic bacteria	Inhibition zone (mm) for various concentrations of MEL-AuNPs		
	25 µg/mL	50 µg/mL	100 µg/mL
*E. coli*	12.5^b^ ± 0.10	16.3^a^ ± 0.11	17.6^a^ ± 0.15
*S. aureus*	12^c^ ± 0.05	14.5^b^ ± 0.05	17^a^ ± 0.18

Different superscript letters in each row indicate significant differences

### Anticancer activity of MEL-AuNPs against the HepG2 cell line

**Fig. 6:**
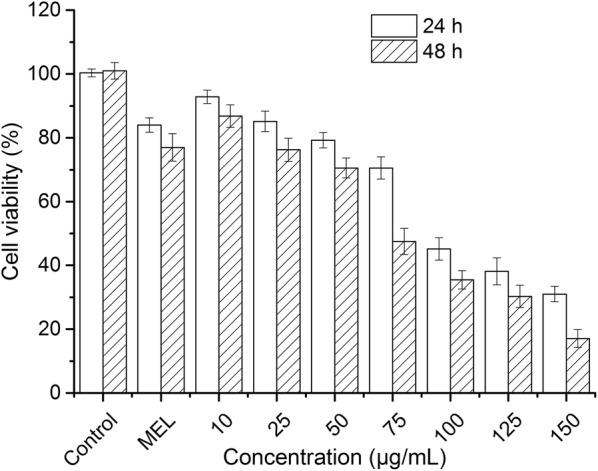
Anticancer activity of the biosynthesized MEL-AuNPs against HepG2 cells after 24 h and 48 h. Each result represented the mean ± standard deviation (SD) and performed in triplicate

The potential cytotoxicity of MEL-AuNPs against human liver cancer cells (HepG2) was examined by the MTT assay using different concentrations (10, 25, 50, 75, 100, 125 and 150 µg/mL) for 24 h and 48 h exposure as shown in Fig. 6. The HepG2 cell population was gradually decreased with an increased MEL-AuNPs concentration and treating time. Considerably, MEL-AuNPs demonstrated potential cytotoxicity on HepG2 cells, the IC_50_ value were 75 and 100 µg/mL for 24 h and 48 h, respectively. The maximum concentration of MEL-AuNPs has inhibited cell growth about 89%.

### Antioxidant capacity of MEL-AuNPs

**Fig. 7:**
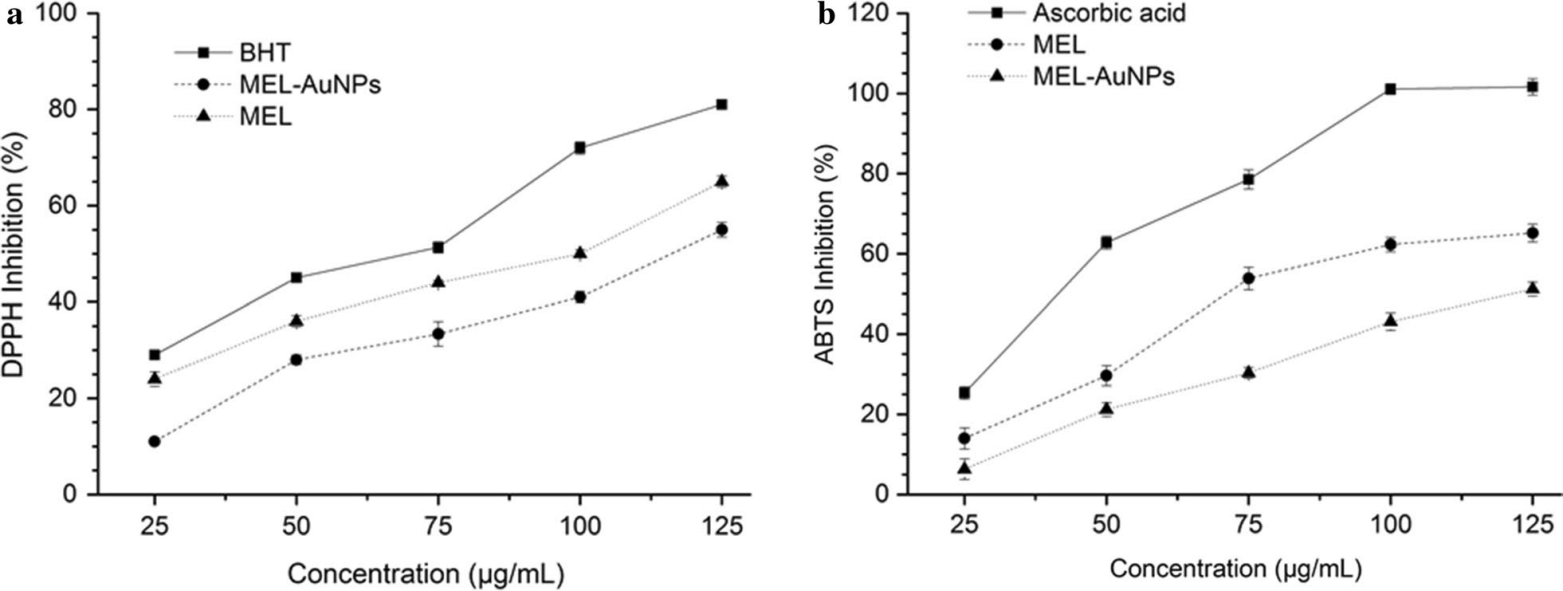
Antioxidant potential of the biosynthesized MEL-AuNPs by use of DPPH and ABTS scavenging evaluation

The antioxidant activity of MEL-AuNPs was evaluated against DPPH and ABTS radicals at various concentrations ranging from 25 to 125 µg/mL. Figure 7a revealed that the DPPH radical scavenging activity was increased with increasing in concentration and showing the highest inhibition of 81%, 65%, and 55% at a maximum concentration corresponding to BHT, MEL, and MEL-AuNPs, respectively. As seen in Fig. 7a, the IC_50_ value of MEL-AuNPs for DPPH activity was found to be 115 µg/mL. The antioxidant capacity of MEL-AuNPs was further investigated by ABTS scavenging using ascorbic acid as a positive control. The MEL-AuNPs showed a dose-dependent activity, and the ABTS scavenging percentages were increased from 6.33% to 51.16%, with an IC_50_ value of about 124 µg/mL (Fig. 7b). We also observed that MEL exhibits superior antioxidant activity than MEL-AuNPs in both assays.

## Discussion

In this study, three types of vegetable oils were used as carbon sources for the production of MEL under growth-limiting nitrogen conditions, using *Ustilago maydis* CGMCC 5.203. The sunflower oil gave a higher yield of MEL than olive and soybean oil. Morita et al. have obtained 2.62 g/L MELs by *U. maydis* NBRC5346 from olive oil (Morita et al. 2009). The present data demonstrated that sunflower oil is the beneficial substrate for MEL formation by *U. maydis* CGMCC 5.203.

Recently, biosurfactants have received growing interest in the area of nanotechnology, owing to their electrostatic force that could uniform the size and shape of particles as well as prevent the aggregation (Kitamoto et al. 2009; Kiran et al. 2011). In the current work, the reduction of HAuCl_4_ to AuNPs was successfully achieved by the aqueous solution of 3 mM HAuCl_4_ and MEL was employed as both the reducing and capping agents in alkaline medium. The strong surface plasmon resonance (SPR) was located at 566 nm, suggesting the fabrication of stable AuNPs by MEL. Typical AuNPs show peaks at wavelengths between 540 to 570 nm (Das et al. 2014; Gómez-Graña et al. 2017; Ramkumar et al. 2017; Singh et al. 2017). There is no noticeable change at the peak after 30 days; we deduced that MEL was acting as an excellent stabilizing agent and hinder the aggregation.

The SEM image shows surface morphology of the biosynthesized MEL-AuNPs in a spherical shape and mostly uniform that may be due to the electrostatic force of biosurfactant MEL. Besides, the TEM image has also confirmed the MEL-AuNPs are mainly composed of spherical and uniform in shape. Furthermore, the EDX Spectrum analysis of MEL-AuNPs proved the existence of Au in the sample, which indicates that Au is the dominant element and confirmed that MELs are capable of synthesizing AuNPs. Comparable peak has been previously reported by (Wang et al. 2016). The crystallinity of MEL-AuNPs was verified by X-ray diffraction technique. The lattice planes of the face-centered cubic (fcc) structure demonstrated that MEL-AuNPs were crystalline. The findings are in accordance with a recent study (Chahardoli et al. 2017).

FTIR spectroscopy was utilized to explore the role of biofunctional groups of MEL that are responsible for the formation of AuNPs. FTIR spectra revealed the possible interactions between the MEL and AuNPs. The bands observed at 3406, 2926–2854, 1743, 1464–1377 and 1171 cm^−1^ corresponding to (O–H), (C–H), (C=O), (C–H) and (C–O), respectively, which are emphasized the structure of MEL, Fukuoka et al. reported similar findings (Fukuoka et al. 2007). The broadband at 3275 cm^−1^ attributed to O–H stretching vibrations. The distinct absorption bands at 1395 cm^−1^ and 2921 cm^−1^ demonstrated the existence of a long fatty acid chain. The strong and broad bands at 2921–2854 cm^−1^ and 1395 cm^−1^ denote the presence of C–H stretching, matching to CH_2_ and CH_3_ groups of aliphatic chains. The absorbance bands at 1635 cm^−1^ and 1179 cm^−1^ indicate the presence of fatty acids. From the results as mentioned above, the existence of fatty acid chain and carbohydrates functional groups represented that it is a MEL composition as well as confirms the interaction between MEL and AuNPs (Gómez-Graña et al. 2017).

In this study, the potential antibacterial activity of MEL-AuNPs displayed a noticeable antibacterial effect against *E. coli* and *S. aureus* at the minimum concentration. Similar results were recently reported by the previous literature (Muthuvel et al. 2014). Previous researchers indicated that the antibacterial activity of nanoparticles facilitated by surfactants, like SDS, Tween 80, PVP 360 and CTAB have significantly improved the antibacterial activity for most species of the bacteria (Kvitek et al. 2008; Alkilany et al. 2009). The supposed mechanism of the antibacterial action by AuNPs is mostly due to; the positive charge of Au^+^ ions linked with the negatively charged bacterial cell wall, inhibits the cellular enzymes and causes disorder in membrane permeability. Additionally, it damages the protein and DNA by releasing reactive oxygen species and thereby leading to cell death (Lee et al. 2003; Sondi and Salopek-Sondi 2004). Besides, nanoparticles with MEL (IL-CS-Nano-MEL) exhibited stronger antibacterial effects against *S. aureus* than IL-CS-Nano, suggesting the antibacterial effects of MEL (Wu et al. 2018).

Moreover, the potential cytotoxicity of MEL-AuNPs on human liver cancer cells (HepG2) was examined by the MTT assay using different concentrations for 24 h and 48 h exposure as shown in Fig. 6. Our findings exhibited a significant inhibitory effect at the low concentration of MEL-AuNPs in comparison with that Muthukumar et al. (2016) has reported an IC_50_ value of 150 µg/mL against HepG2 cells for AuNPs prepared by *Catharanthus roseus* leaf extract. According to the several reports, The cytotoxicity of AuNPs was ascribed to the interactions between cells and NPs, which relied on physiochemical properties of NPs such as size and shape of particles, surface charge, the nature of cells, the NPs concentration and treating time (Lee et al. 2015; Muthukumar et al. 2016; Chahardoli et al. 2017).

Finally, the antioxidant activity of MEL-AuNPs was evaluated against DPPH and ABTS radicals at various concentrations. Figure 7a, b revealed that the DPPH and ABTS radical scavenging activity was increased with increase in concentration. The IC_50_ value of MEL-AuNPs for DPPH and ABTS activity was found to be 115 µg/mL and 124 µg/mL, respectively. The results thus obtained are compatible with that Muthuvel et al. (2014) have got a similar effect on DPPH activity by AuNPs synthesized using *Solanum nigrum* leaf extract. We also observed that MEL exhibits superior antioxidant activity than MEL-AuNPs in both assays. This results might be possible due to that the unsaturated fatty acids in MEL lead to increase the antioxidant activity by working as donors of hydrogen atoms (Takahashi et al. 2012).

In summary, a promising mannosylerythritol lipids (MELs) produced from *U. maydis* CGMCC 5.203 was utilized as green reducing/stabilizing agents to synthesize AuNPs. Based on the results, we concluded that the biosynthesized MEL-AuNPs had good physical characteristic and proved potential bioactivities such as antibacterial activity against pathogenic bacteria, antioxidant, and toxicity against HepG2 cells. The present data can be used for diagnosis and cancer therapy as drug delivery as well as an antibiotic compound.
